# RICTOR involvement in the PI3K/AKT pathway regulation in melanocytes and melanoma

**DOI:** 10.18632/oncotarget.4866

**Published:** 2015-08-27

**Authors:** Florence Laugier, Adeline Finet-Benyair, Jocelyne André, P. Sivaramakrishna Rachakonda, Rajiv Kumar, Armand Bensussan, Nicolas Dumaz

**Affiliations:** ^1^ INSERM, U976, Centre de Recherche sur la Peau, Hôpital Saint-Louis, Paris, F-75010, France; ^2^ Université Paris Diderot, Sorbonne Paris Cité, UMRS976, Paris, F-75010, France; ^3^ Division of Molecular Genetic Epidemiology, German Cancer Research Center, 69120 Heidelberg, Germany

**Keywords:** RICTOR, PI3K, AKT, melanoma, mTORC2

## Abstract

Several studies have highlighted the importance of the PI3K pathway in melanocytes and its frequent over-activation in melanoma. However, little is known about regulation of the PI3K pathway in melanocytic cells. We showed that normal human melanocytes are less sensitive to selective PI3K or mTOR inhibitors than to dual PI3K/mTOR inhibitors. The resistance to PI3K inhibitor was due to a rapid AKT reactivation limiting the inhibitor effect on proliferation. Reactivation of AKT was linked to a feedback mechanism involving the mTORC2 complex and in particular its scaffold protein RICTOR. RICTOR overexpression in melanocytes disrupted the negative feedback, activated the AKT pathway and stimulated clonogenicity highlighting the importance of this feedback to restrict melanocyte proliferation. We found that the RICTOR locus is frequently amplified and overexpressed in melanoma and that RICTOR over-expression in NRAS-transformed melanocytes stimulates their clonogenicity, demonstrating that RICTOR amplification can cooperate with NRAS mutation to stimulate melanoma proliferation. These results show that RICTOR plays a central role in PI3K pathway negative feedback in melanocytes and that its deregulation could be involved in melanoma development.

## INTRODUCTION

The signaling pathway defined by phosphoinositide 3-kinase (PI3K), AKT and mammalian target of rapamycin (mTOR) controls most hallmarks of cancer, including cell cycle, survival, metabolism, motility and genomic instability [1]. Cancer genetic studies have shown that this pathway is among the most frequently altered pathway in human tumors [2]. In melanoma, multiple lines of evidence have shown that the PI3K pathway plays a major role in the pathogenesis of this disease, and cooperates with activation of the RAS–RAF–MEK–ERK pathway to transform melanocytes [3, 4].

PI3K can be activated by multiple signals, including Receptor Tyrosine Kinases (RTKs) and RAS proteins among others. Activated PI3K phosphorylates phosphatidylinositol-4,5-biphosphate (PIP2) to phosphatidylinositol-3,4,5-triphosphate (PIP3), which in turn attracts proteins that contain a pleckstrin homology domain to the cell membrane, including the serine-threonine kinase AKT and phosphoinositide-dependent kinase-1 (PDK1). AKT, which has three isoforms (AKT1/2/3), is phosphorylated at two critical and conserved residues, which activate its catalytic activity. One residue is in the kinase domain (threonine 308 [Thr308]) and the other in the hydrophobic motif (serine 473 [Ser473]). Thr308 is phosphorylated by membrane localized PDK1, and Ser473 by the mammalian target of rapamycin complex 2 (mTORC2). RICTOR is a component of the mTORC2 complex which is required for AKT Ser473 phosphorylation [5]. mTORC2 is involved in cell survival [5–7], actin cytoskeleton remodeling [8, 9] and glucose transport [10]. Activated AKT phosphorylates a number of effector proteins, thereby regulating multiple key cellular processes, including proliferation, survival, motility, metabolism and angiogenesis. AKT plays a critical role in survival through the inhibition of BAD [11, 12] as well as the regulation of cell cycle entry by inactivating glycogen-3 synthase kinase (GSK3), leading to the activation of cyclin D1 [13]. AKT, through TSC2 phosphorylation and RHEB activation [14], also activates mTORC1 which is involved in ribosomal biogenesis and protein synthesis via phosphorylation of S6K and 4EBP1. PTEN regulates the activity of this pathway by dephosphorylating phosphatidylinositols, thereby antagonizing the activity of PI3K [15].

A large number of studies have shown that the PI3K pathway is frequently over-activated in melanoma via multiple ways. The two most common and studied events are activating mutations in the oncogene NRAS (15–20%) [16–18], and loss of expression or function of the tumor suppressor PTEN (11–60%) [16, 19, 20–24], but mutations of PI3K [25, 26] and AKT3 [27], the predominant AKT isoform in melanoma [28], have also been described. Increased AKT activity is also a hallmark of melanoma resistance to targeted therapy [29–34]. The central role of PI3K activation in tumor cell biology has prompted a considerable effort to target PI3K or downstream kinases, such as AKT and mTOR, in cancer. However, emerging clinical data show limited single-agent activity of inhibitors targeting PI3K, AKT or mTOR at tolerated doses [35–37]. The development of effective strategies against this pathway must overcome several key challenges, in particular feedback loops, and pathway cross-talk that can mediate resistance. Feedback control is a common feature of cellular signaling systems, and the PI3K network provides many examples of complex feedback perturbations generated by PI3K, AKT and mTOR inhibitors [38]. The best example is the feedback induction of AKT phosphorylation by mTORC1 inhibitors, such as rapamycin [39, 40]. To overcome complex feedback loops that blunt the activity of single-target inhibitors against the PI3K–AKT pathway and to achieve effective clinical targeting of melanoma, it is essential to better understand the complex regulation of the PI3K pathway in melanocytes and melanoma.

In this paper we investigate the regulation of the PI3K pathway in melanocytes and melanoma. We showed that normal human melanocytes are less sensitive to selective PI3K or mTOR inhibitors than to dual PI3K/mTOR inhibitors. The resistance to PI3K inhibitors is due to a rapid AKT reactivation following inhibitor treatment, linked to a feedback mechanism involving RICTOR, a scaffold protein of the mTORC2 complex. RICTOR overexpression in melanocytes activates the PI3K pathway and stimulates clonogenicity. We found that the RICTOR locus is frequently amplified and overexpressed in melanoma and that RICTOR over-expression in melanoma stimulates clonogenicity. These results show that RICTOR plays a central role in PI3K pathway negative feedback in melanocytes and that its deregulation could be involved in melanoma development.

## RESULTS

### Melanocytes are more sensitive to dual PI3K/mTOR inhibition than PI3K or mTORC1 inhibition

**Figure 1:**
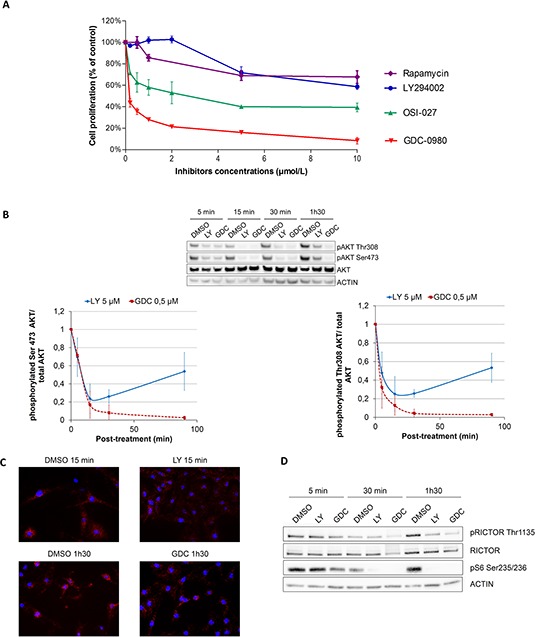
Melanocytes are more sensitive to dual PI3K/mTOR inhibition than PI3K inhibition due to a feedback mechanism inducing AKT reactivation **A.** NHEMs were treated for 3 days with different concentrations of LY294002, OSI-027 or GDC-0980 in growing medium and proliferation was analyzed with CellTiter (data are represented as mean +/− SD). **B.** NHEMs were treated for 5, 15, 30, 90 minutes with DMSO (control), 5 μM LY294002 or 0.5 μM GDC-0980. Levels of phosphorylated proteins and total proteins were analyzed by Western Blotting (data are represented as mean +/− SD). **C.** Melan-a cells were treated for 15 min or 1 h30 with LY294002 or GDC-0980 and interaction between RICTOR and AKT was studied by proximity ligation assay. **D.** NHEMs were treated for 5, 15, 30, 90 minutes with DMSO (control), 5 μM LY294002 or 0.5 μM GDC-0980. Levels of phosphorylated protein or total protein were analyzed by Western Blotting.

To address the role of the PI3K pathway in melanocyte proliferation, Normal Human Epidermal Melanocytes (NHEMs) and immortal mouse melanocytes (Melan-a) were treated with increasing doses of mTORC1 inhibitor (Rapamycin), PI3K inhibitor (LY294002), mTORC1/mTORC2 inhibitor (OSI-027) or dual PI3K/mTOR inhibitor (GDC-0980). LY294002 treatment did not significantly inhibit melanocyte proliferation at 2 μmol/L (Figure 1A and Supplementary Figure S1). However, inhibition of both PI3K and mTOR complexes (mTORC1 and mTORC2) by GDC-0980 led to a very strong inhibition of melanocyte proliferation at low concentration (0.5 μmol/L) (Figure 1A and Supplementary Figure S1). To confirm this result, another selective PI3K inhibitor (ZST474) with an IC50 for PI3K similar to GDC-0980 was used. Melanocyte proliferation was still less sensitive to ZSTK474 than to GDC-0980 (Supplementary Figure S1). Inhibition of mTORC1 by rapamycin or inhibition of both mTORC1 and mTORC2 by OSI-027 had a weaker effect than the combined inhibition of PI3K and mTOR by GDC-0980 (Figure 1A and Supplementary Figure S1). Therefore, the PI3K pathway and in particular mTORC1 and mTORC2 seems to play an important role in both human and murine melanocyte proliferation.

To understand the molecular mechanism of resistance to PI3K pathway inhibitor, inhibition of the PI3K pathway was monitored in human melanocytes and Melan-a by western blotting using AKT phosphorylation as a marker of pathway activation. Both LY294002 and GDC-0980 induced a significant inhibition of AKT phosphorylation at both activation sites (Ser473 and Thr308) after 5 to 30 minutes of treatment (Figure 1B and Supplementary Figure S2). However, AKT phosphorylation increased again after 15 min of treatment with LY294002 treatment, whereas it remained inhibited up to 90 min in GDC-0980 treated melanocytes (Figure 1B and Supplementary Figure S2). A similar reactivation of AKT was seen after 6 hours of treatment with another selective PI3K inhibitor (ZSTK 474) but not with GDC-0980 (Supplementary Figure S3). These results suggested the existence of a feedback mechanism downstream of PI3K and dependent on mTOR complexes that could stimulate AKT reactivation. The rapid reactivation of AKT upon LY294002 treatment could explain the low efficiency of this inhibitor on melanocyte proliferation.

Several feedback mechanisms in the PI3K pathway have been described. Among them, we further investigated the feedback mechanism implicating RICTOR, a scaffold protein from the mTORC2 complex, because it had been suggested that RICTOR could play a role in cancer [7, 41].

We first showed, using proximity ligation assay, that RICTOR interacts with AKT in melanocytes and that this interaction is independent of AKT phosphorylation, as it is not modulated by GDC-0980 which inhibits AKT phosphorylation (Figure 1C and Supplementary Figure S4).

RICTOR is regulated by phosphorylation at threonine 1135 (Th1135) by S6K, downstream of PI3K, which inactivates mTORC2 and therefore inhibits AKT phosphorylation at Ser473 [42–44]. We showed that RICTOR is phosphorylated on Thr1135 in melanocytes and that both PI3K inhibitors, LY294002 and GDC-0980, decreased Thr1135 phosphorylation (Figure 1D). This decrease is linked to an inhibition of S6K activity, measured by a decrease in phosphorylation of its target: the ribosomal protein S6 at Ser235/236, in response to LY294002 and GDC-0980 treatment (Figure 1D). This inhibition of RICTOR phosphorylation, which will activate mTORC2, could be responsible for AKT reactivation in the presence of PI3K inhibitor.

### RICTOR controls the PI3K pathway in melanocytes

**Figure 2:**
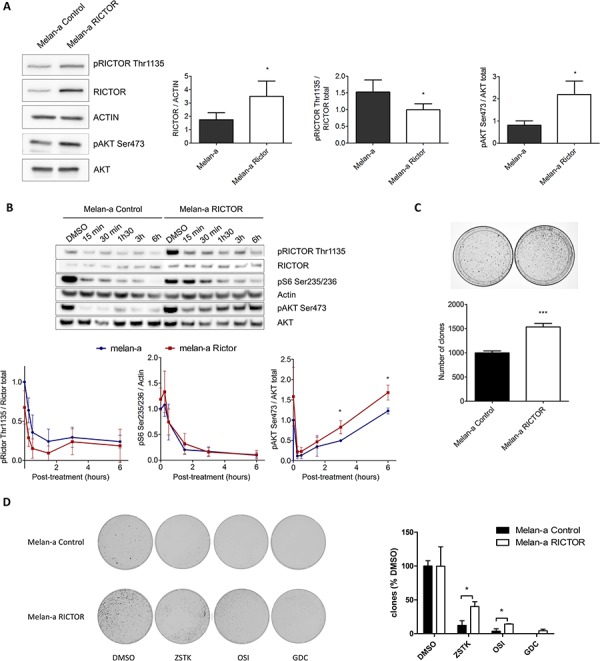
RICTOR controls the PI3K pathway in melanocytes **A.** Melan-a were transfected with empty vector or with vector containing human RICTOR cDNA. Levels of phosphorylated proteins and total proteins were analyzed by Western Blotting (data are represented as mean +/− SD). **B.** Melan-a and Melan-a overexpressing RICTOR were treated with DMSO or LY294002 at 5 μM for 15 min, 30 min, 1 h30, 3 h or 6 h (data are represented as mean +/− SD). **C.** Melan-a were transfected with empty vector or with vector containing human RICTOR cDNA. Cells were selected and colonies counted after 10 days (data are represented as mean +/− SD). **D.** After transfection with empty vector or with vector containing human RICTOR cDNA, Melan-a were selected by resistance to blasticidin for 3 days. Then in addition to blasticidin selection, Melan-a were treated twice a week with ZSTK474, OSI-027, GDC-0980 at respectively 0.2 μM, 5 μM and 0.5 μM or DMSO as control. After 2 weeks colonies were counted (data are represented as mean +/− SD).

To understand the role of RICTOR in AKT reactivation, we overexpressed RICTOR in Melan-a. Melan-a overexpressing RICTOR (Melan-a RICTOR) displayed a reduced ratio of phosphorylated RICTOR over total RICTOR and a higher level of phosphorylated AKT at Ser473 compared to control (Figure 2A). LY294002 treatment induced similar inhibition of RICTOR Thr1135 and S6 Ser235/236 phosphorylations in Melan-a RICTOR and control melanocytes (Figure 2D). Although LY294002 treatment induces the same level of AKT phosphorylation inhibition in Melan-a RICTOR and in control, there was a higher reactivation of AKT phosphorylation in Melan-a RICTOR compared to control melanocytes (Figure 2B). As expected, we showed that treatment with GDC-0980, the dual PI3K/mTOR inhibitor, led to stable inhibition of RICTOR, S6 and AKT phosphorylations in both Melan-a RICTOR and control (Supplementary Figure S2). Therefore, we can assume that lower level of phosphorylated RICTOR Thr1135 reduces the self-regulation of the pathway and allows a stronger reactivation of AKT Ser473. These results showed that RICTOR overexpression disrupted the complex regulation of the PI3K pathway and supported a role for RICTOR in a feedback mechanism upon PI3K inhibition in melanocytes.

To investigate the biological effect of RICTOR overexpression in melanocytes, we compared the clonogenicity of Melan-a RICTOR to control Melan-a. We showed that RICTOR overexpression induced a statistically significant increase in Melan-a colony formation (Figure 2C), demonstrating that disrupting the PI3K pathway in melanocytes by overexpressing RICTOR, stimulated their clonogenicity. The clonogenic assay was reproduced in the presence of PI3K, mTORC1/mTORC2 or dual PI3K/mTOR inhibitors. RICTOR overexpression induced resistance to the PI3K inhibitor compared to the mTORC1/mTORC2 or dual PI3K/mTOR inhibitors (Figure 2D).

### RICTOR locus is amplified in melanoma and deregulates the PIK pathway

**Figure 3:**
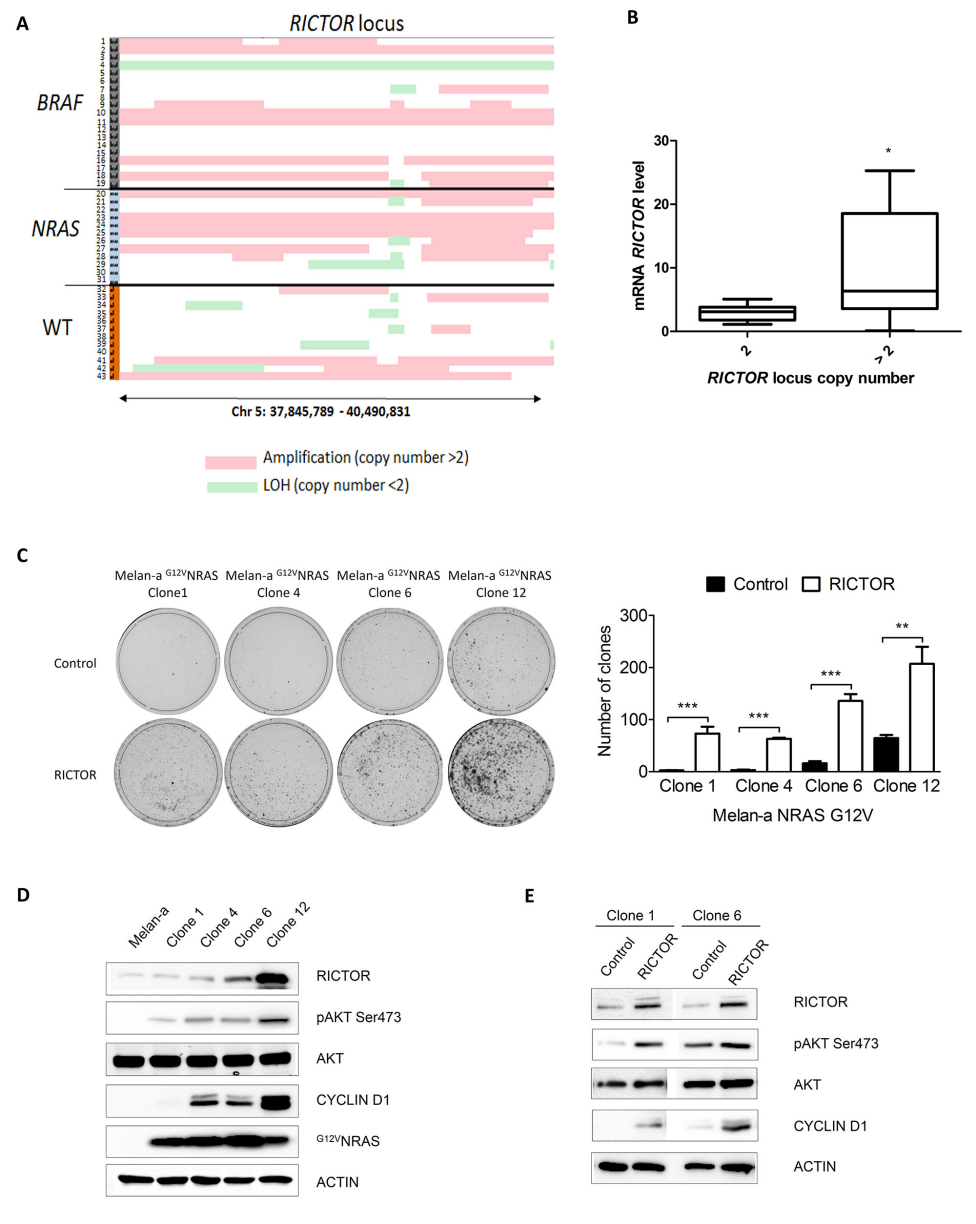
RICTOR locus is amplified in melanoma and stimulates clonogenicity and cyclinD1 expression in ^G12V^NRAS transformed Melan-a **A.** Melanoma cell lines derived from patients were analyzed by CGH array at *RICTOR* and *PTEN* loci. Numbers 1 to 43 indicate cell line number. Red lines correspond to amplification (copy number > 2) and green lines to loss of heterozygosity (copy number < 2). **B.** RICTOR mRNA level was quantified by quantitative RT-PCR in 22 short term melanoma cell lines with (> 2) or without (2) *RICTOR* locus amplification. **C.** Transformed ^G12V^NRAS Melan-a were transfected with empty vector or with vector containing human RICTOR cDNA. Cells were selected by blasticidin and colonies were counted after 10 days (data are represented as mean +/− SD). **D.** Levels of phosphorylated proteins and total proteins were analyzed by Western Blotting in 4 different clones of transformed ^G12V^NRAS Melan-a (clone 1, 4, 6 and 12). **E.** Transformed ^G12V^NRAS Melan-a clones 1 and 6 were transfected with empty vector or with vector containing human RICTOR cDNA and levels of phosphorylated proteins and total proteins were analyzed by Western Blotting.

As RICTOR overexpression increases melanocyte clonogenicity, we investigated whether RICTOR amplification could play a role in melanoma. Using CGH array, we analyzed the amplification of the RICTOR locus in a series of 43 melanoma short-term cultures. We found that the *RICTOR* locus was amplified in 19 out of 43 melanoma cell lines (44%) and that amplification was independent of the BRAF and NRAS mutation status (Figure 3A and Supplemenentary Data). Quantification of RICTOR mRNA in 22 melanoma short-term cultures confirmed that RICTOR locus amplification was associated with an increase in RICTOR mRNA level (Figure 3B). RICTOR amplification and PTEN loss of heterozygosity (LOH) were not mutually exclusive and in BRAF mutated cell lines amplification at the *RICTOR* locus were always associated with LOH at *PTEN* locus (Supplementary Figure S5). RICTOR amplification is therefore a frequent event in melanoma and can be associated with PTEN loss.

To investigate whether RICTOR amplification could play a role in melanoma development, we stably overexpressed RICTOR in Melan-a that had been transformed by ^G12V^NRAS. We used four different transformed clones expressing different levels of endogenous RICTOR but with the same genetic background allowing us to compare the clones exclusively at the RICTOR level. RICTOR overexpression induced a statistical increase in colony formation in all four clones (Figure 3C). Clones overexpressing RICTOR displayed an increase in AKT phosphorylation demonstrating that RICTOR overexpression can activate the PI3K/AKT pathway in melanoma (Figure 3E). This increase in AKT phosphorylation was associated with an increase in cyclin D1, which could explain the increase in colony formation (Figure 3E). Interestingly, clones expressing higher levels of endogenous RICTOR (6 and 12) showed a higher number of colonies with the vector control compared to the clones expressing lower levels of endogenous RICTOR (1 and 4) (Figure 3C and 3D). The effect of RICTOR overexpression on colony formation was less pronounced in clones expressing high levels of endogenous RICTOR (6 and 12) compared to clones expressing low levels of endogenous RICTOR (1 and 4) (Figure 3C and 3D). Moreover, the level of cyclin D1 protein was proportional to the level of RICTOR in the clones (Figure 3D) suggesting a role for RICTOR in cyclin D1 expression and melanoma proliferation.

**Figure 4:**
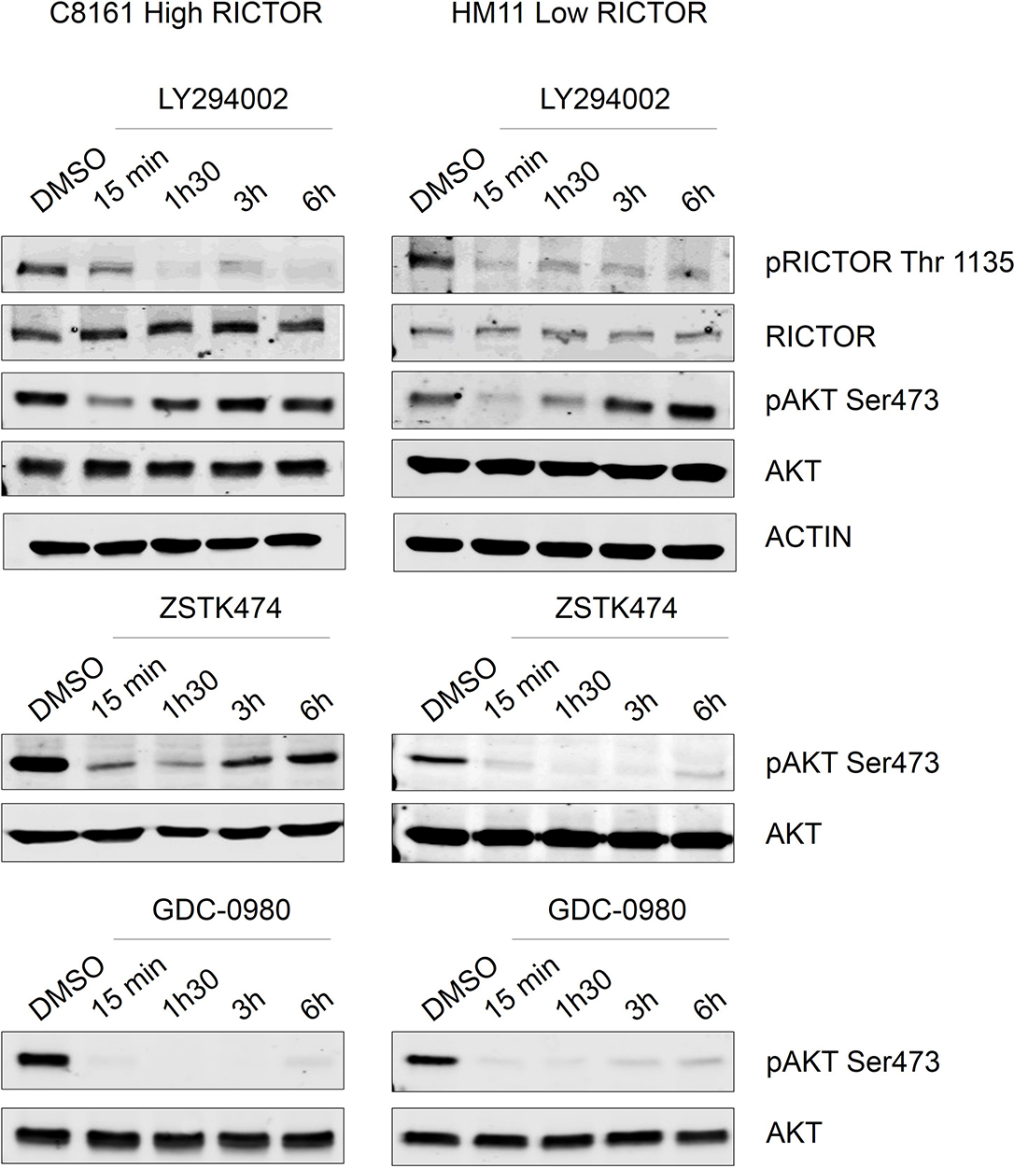
Reactivation of AKT is increased in ^G12V^NRAS mutated melanoma cell line expressing high level of RICTOR compared low level of RICTOR Human ^Q61K^NRAS mutated melanoma cell lines, C8161, expressing a high level of RICTOR, and HM11, expressing a low level of RICTOR were treated for 15 minutes, 1 h30, 3 hours or 6 hours with DMSO (control), LY294002 (5 μM), ZSTK474 (0.2 μM) or GDC-0980 at (0.5 μM). Level of phosphorylated protein or total protein are analyzed by Western Blotting.

To confirm these results in human melanoma cells, we used two NRAS mutated melanoma cell lines which express different levels of RICTOR. We quantified the ratio RICTOR/AKT and showed that C8161 (High-RICTOR) expressed 6 times more RICTOR than HM11 (Low-RICTOR) (Figure 4). As shown for ^G12V^NRAS transformed Melan-a cells, human melanoma cells expressing a high level of RICTOR showed a higher level of phosphorylated AKT (Figure 4). Moreover a strong reactivation of AKT phosphorylation was seen following treatment with two selective PI3K inhibitors in High-RICTOR cells whereas AKT reactivation was much weaker in Low-RICTOR cells (Figure 4). As expected, the treatment with GDC-0980, the dual PI3K/mTOR inhibitor, led to stable inhibition of AKT phosphorylation in both High-RICTOR and Low-RICTOR melanoma cells (Figure 4). These results confirmed that RICTOR overexpression disrupts the regulation of the PI3K pathway in human melanoma cells.

## DISCUSSION

To improve currently available melanoma treatment, we need to identify and target the melanocyte-specific molecular and cellular events involved in the initiation and progression of melanocytes towards melanoma. In particular, we ought to decipher the signaling pathways, which play a major role in melanocytes and melanoma in order to find novel therapeutic targets. Genetics studies and the use of small molecule inhibitors have demonstrated the importance of the PI3K pathway in melanocytes, however, little is known about regulation of the PI3K pathway in these cells. We showed here that inhibition of the PI3K/AKT/mTOR pathway induced strong inhibition of melanocyte proliferation, highlighting the important role of PI3K pathway in melanocytes, which has previously been shown during melanocyte development in mouse and *Xenopus laevi* [45].

Although melanocytes were very sensitive to a dual PI3K/mTOR inhibitor, they were less sensitive to a sole PI3K inhibitor due to a feedback mechanism leading to rapid reactivation of AKT. Several feedback mechanisms have been described in the PI3K pathway and the most studied one is the feedback mechanism associated with rapamycin treatment. Rapamycin induced a strong reactivation of AKT for at least 24 hours (Supplementary Figure S3). Inhibition of mTORC1 by rapamycin down regulates the feedback inhibition of S6K on IRS-1 an adaptor protein activating PI3K, which will eventually lead to AKT reactivation [40, 46]. However, in our study with PI3K inhibitors, inhibition of PI3K prevents this feedback mechanism to reactivate AKT. Instead, we show that reactivation of AKT involves mTORC2 and more precisely RICTOR, a scaffold protein which plays an essential role in mTORC2 activity. It was described in HEK-293, HeLa and MEF cells, that the p70 ribosomal S6 kinase (S6K), downstream of mTORC1, phosphorylates RICTOR at Thr1135, inhibiting mTORC2 activity and hence AKT Ser473 phosphorylation [42–44]. How phosphorylation of RICTOR Thr1135 inhibits AKT Ser473 phosphorylation is not yet fully understood but we showed that AKT is linked to RICTOR in melanocytes and that this interaction is not altered by RICTOR Thr1135 phosphorylation. Dibles et al. have already shown that mTORC2 assembly is not altered by RICTOR phosphorylation but that RICTOR phosphorylated on Thr1135 binds to 14–3-3, inducing a conformational change that prevents mTORC2 from phosphorylating AKT [42]. We further showed that over expression of RICTOR in Melan-a disrupted this feedback mechanism, enhancing AKT activation and stimulating Melan-a clonogenicity. RICTOR overexpression also induced resistance to a selective PI3K inhibitor. These results suggest that this feedback mechanism is important to restrict melanocyte proliferation and that over expression of RICTOR could participate in melanoma progression by enhancing the PI3K pathway. Indeed, we found that the locus coding for *RICTOR* is frequently amplified in melanoma independently of BRAF or NRAS mutations and that RICTOR amplification in BRAF mutated melanoma is associated with PTEN LOH. *RICTOR* locus amplification is associated with a significant increase in RICTOR mRNA expression in melanoma. Although RICTOR overexpression was not oncogenic in Melan-a, overexpression of RICTOR in transformed ^G12V^NRAS Melan-a stimulated their clonogenicity, demonstrating that RICTOR amplification can cooperate with NRAS mutation to stimulate melanoma proliferation. The effect of RICTOR on proliferation could be linked to its effect on cyclin D1 expression as we showed that the level of RICTOR in transformed ^G12V^NRAS Melan-a is correlated to the level of cyclin D1 and to cellular proliferation. Overexpression of RICTOR in transformed ^G12V^NRAS Melan-a further enhanced cyclin D1 expression and cellular proliferation. The effect of RICTOR on cyclin D1 could be linked to its activation of AKT and the subsequent inhibition of GSK3. GSK3 phosphorylates cyclin D1 and induces its degradation but it is inhibited by AKT phosphorylation. AKT activation due to RICTOR overexpression could therefore stabilize cyclin D1 by inhibiting its GSK3 dependent phosphorylation. Further experiments are needed to decipher the link between RICTOR and cyclin D1. RICTOR amplification, by stimulating AKT phosphorylation, may also act on other AKT effectors. Indeed, although many studies have shown that phosphorylation of AKT at both Ser473 and Thr308 are needed for AKT full activation [47], we know now that phosphorylation of AKT at Ser473 is not needed for mTORC1 activation and by consequence for protein synthesis and cell growth, whereas phosphorylation of AKT at Thr308 is essential for these activities [5]. On the other hand, phosphorylation of AKT at Ser473 is essential for cell survival and glucose uptake [5–7, 10]. This could explain why RICTOR overexpression in Melan-a induced a higher number of colonies as a consequence of increased survival rather than an enlargement of colonies, which is a consequence of increased cell growth. In accordance with this, Melan-a RICTOR do not display increased proliferation compared to parental cells (data not shown). The effect of RICTOR overexpression on clonogenicity may also be linked to other targets independent of mTORC2 because Melan-a RICTOR melanocytes are more resistant than parental cells to the mTORC2 inhibitor OSI-027. Further experiments are needed to identify RICTOR targets involved in this process.

Our results demonstrated that RICTOR cooperates with oncogenic NRAS by increasing AKT activation and stimulating clonogenicity of melanoma cells. The CGH data showing that *RICTOR* is also amplified in BRAF mutated melanoma, suggest that RICTOR could also cooperate with oncogenic BRAF. However, *RICTOR* amplification is always associated with *PTEN* LOH in BRAF mutated melanoma, suggesting that RICTOR overexpression may not enhance AKT activation in the presence of PTEN in these melanoma. This effect could be due to the fact that ^V600E^BRAF, but not wild type BRAF, has been found to inhibit mTORC2 induced phosphorylation of AKT Ser473 in the presence of PTEN [48]. However, deletion of PTEN, activates AKT and overrides ^V600E^BRAF induced inhibition of AKT [48]. Therefore, if mTORC2 activity is inhibited by ^V600E^BRAF in PTEN wild type melanoma, RICTOR overexpression will not enhance AKT activation in these cells. In agreement with this hypothesis, we found that RICTOR overexpression does not enhance clonogenicity of the SkMel5 melanoma cell lines, which is ^V600E^BRAF mutated and PTEN wild type (data not shown).

In conclusion, we showed that RICTOR is responsible for an important negative feedback in the PI3K pathway in melanocytes, restricting their proliferation. Activation of the PI3K/AKT pathway is frequent in melanoma and may enhance this negative feedback, so malignant cells will have to find a way to override these feedback, for example by overexpressing RICTOR. Amplification of the *RICTOR* locus, which is frequently found in melanoma, disrupts this feedback and stimulates melanocyte and melanoma proliferation. Although we do not know yet whether *RICTOR* amplification is an early or late event in melanoma development, it is more likely to be a secondary event to reinforce AKT pathway activation. Our results highlight the importance of RICTOR in melanoma in agreement with Werzowa et al., who have previously shown that silencing of RICTOR reduced melanoma cell line viability and increased apoptosis [41]. The same group has shown that vertical inhibition of the PI3K pathway leads to an enhanced reduction of melanoma tumor growth [49]. These findings show the importance of targeting the PI3K/AKT/mTOR pathway at several points in melanoma to prevent feedback.

## MATERIALS AND METHODS

### Plasmid and reagents

Myc epitope–tagged RICTOR was obtained from Addgene and subcloned in the PEF6/V5-His-TOPO^®^ vector (Invitrogen). LY294002 and ZSTK474, PI3K inhibitors, GDC-0980 (RG7422), PI3K and mTOR inhibitor and OSI-027, mTORC1 and mTORC2 inhibitor, were obtained from Selleckchem.

### Cell culture

Melan-a cell line, a non transformed mouse melanocyte line that retains many of the characteristics of normal melanocytes [50] was cultured in RPMI 1640 (Invitrogen) containing 10% (v/v) fetal calf serum (FCS; Perbio), L-glutamin (2 mM; Invitrogen), antibiotics (100 U/ml penicillin and 1000 μg/ml streptomycin; Invitrogen), 100 nM TPA (Sigma), and 200 pM cholera toxin (Sigma). NHEMs (Promo Cell) were cultured in KBM-Gold medium containing 0.5% serum and SCF (Lonza).

For *ex vivo* culture, Melan-a cells and NHEMs both require ERK signaling to stimulate proliferation and cAMP signaling to maintain their differentiated phenotype. Thus, they are both cultured in a medium supplemented with 12-O-Tetradecanoylphorbol 13-acetate (TPA) to stimulate ERK signaling and choleric toxin (Melan-a) or α-MSH (for NHEMs) to stimulate cAMP signaling.

### Melan-a transfection and cloning assay

Melan-a cells were transfected in 6-well plate (400000 cells/well) with JetPEI (Polyplus-transfection) according to the manufacturer's instructions. Cells were selected by blasticidin (10 μg ml^−1^; PAA) and either fixed and stained with 0.5% (v/v) crystal violet + 20% methanol or collected and grown up for further analysis.

Melan-a ^G12V^NRAS clones grew in complete RPMI medium (without TPA and cholera toxin) and were transfected in 6-well plate (300000 cells/well) with lipofectamine 2000 according to manufacturer instructions. After 2 days cells are counted and moved in a 10 cm culture dish and selected by blasticidin (10 μg ml^−1^; PAA). Forming colonies are then stained with 0.5% (v/v) crystal violet+ 20% methanol or collected and grown up for further analysis.

### MTS proliferation assay

Melan-a or NHEMs were dispensed into 96-well plates at 10000 or 1000 cells, respectively, in 180 μl of growing medium (detailed in “cell culture” section). 20 μl of inhibitor solution at the appropriate concentration were added. After three days of treatment, proliferation was measured using 20 μl per well of CellTiter 96^®^Aqueous One Solution Reagent (Promega).

### Western blotting

Cells were lysed in 50 mM Tris, pH 7.5, 150 mM NaCl, 0.5% (v/v) NP-40, 5 mM NaF, 2 mM Na_3_VO_4_, 10 μg ml^−1^ leupeptin and 10 μg ml^−1^ aprotinin. 20–25 μg of proteins were separated by SDS-PAGE and western blot analysis was carried out according to standard protocols using the following antibodies: phosphoAKT (ser473) XP, phosphoAKT (thr308), AKT, phosphoRICTOR (thr1135), RICTOR, phosphoS6 (ser235/236; Cell Signaling Technology) and Actin (Abcam). Proteins were revealed and quantified with SuperSignal^®^ West Pico Chemiluminescent Substrate (Thermo Scientific) on an ImageQuant imaging system or by fluorescence on an Odyssey imaging system.

### Proximity ligation assay

After treatment with inhibitors or DMSO cells were fixed with 4% paraformaldehyde for 15 min, then permeabilized with 0, 5% Triton X100. DuoLink assay (OLINK BIOSCIENCE) was performed according to manufacturer instructions using AKT and RICTOR antibodies.

### Array-based comparative genomic hybridization (arrayCGH)

We utilized previously obtained CGH array data from 44 cell lines and corresponding peripheral blood mononuclear cells (PBMC) to determine copy number changes at the *RICTOR* and *PTEN* loci [51]. A 2.6 Mb region covering 236 SNPs in *RICTOR* gene locus (Chr 5:37,845,789–40,490,831) and a 2.1 Mb region covering 150 SNPs in *PTEN* locus (Chr 10:88,570, 230–90,707,272) were analyzed for amplifications and loss of heterozygosity (LOH) between the three groups of cell lines that contained either BRAF, NRAS mutations or did not carry mutations in either of the two genes. Pair wise copy number (CN) analysis and LOH at the *RICTOR* and *PTEN* loci was carried out using chromosome copy number analysis tool (CNAT 4.0, Affymetrix). The copy number data was exported into Excel (see supplementary data) and SNPs that showed > 2 copies were colored red (amplifications) and the SNPs < 2 were colored green (LOH). Chi-square test was carried out between cell lines without mutations and cell lines with either BRAF or NRAS mutations for amplifications at RICTOR and LOH at PTEN loci.

### Quantitative reverse transcription-PCR

RNA was extracted from short term melanoma cultures and reverse transcribed. RICTOR expression levels were determined by quantitative real-time PCR. PCR reactions were done in duplicate using the Power SYBR Green PCR Master Mix kit (Applied Biosystems) following the manufacturer's instructions, using forward (CCGTGTCGGAGGTTCATACA) and reverse (GCCTCTGCTTCTTCATGCATT) RICTOR primers and forward (TGGGTGTGAACCATGAGAAGTATG) and reverse (GGTGCAGGAGGCATTGCT) GADPH primers, a housekeeping gene used as an internal standard. RICTOR expression levels were calculated using GADPH expression as a reference and relative quantification was performed using the ΔΔ Ct method and log2 transformation.

### Statistical analysis

Data show the mean of at least three independent experiments. GraphPad Prism statistical tool was used to perform the Student's *t*-test where ns > 0.05, *P < 0.05, **P < 0.01, ***P < 0.001 and ****P < 0.0001.

## SUPPLEMENTARY FIGURES AND SUPPLEMENTARY DATA




