# Supplementary material for: RICTOR involvement in the PI3K/AKT pathway regulation in melanocytes and melanoma
Source: Oncotarget. 2015 Aug 27;6(29):28120–31. doi: 10.18632/oncotarget.4866 (PMC4695048; doi:10.18632/oncotarget.4866)
Supplement: Supplementary file 1 [file oncotarget-06-28120-s001.pdf]

SUPPLEMENTARY FIGURES AND SUPPLEMENTARY DATA

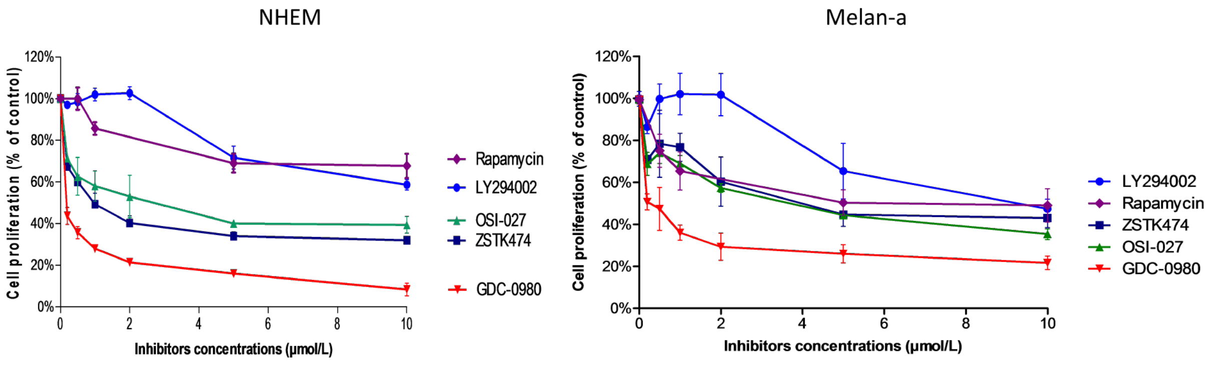

Supplementary Figure S1: Melanocytes are more sensitive to dual PI3K/mTOR inhibition than single PI3K inhibition. NHEM or Melan-a were treated for 3 days with different concentrations of LY294002, ZSTK474, rapamycin, OSI-027 or GDC-0980 in their respective growing medium and proliferation was analyzed with CellTiter.

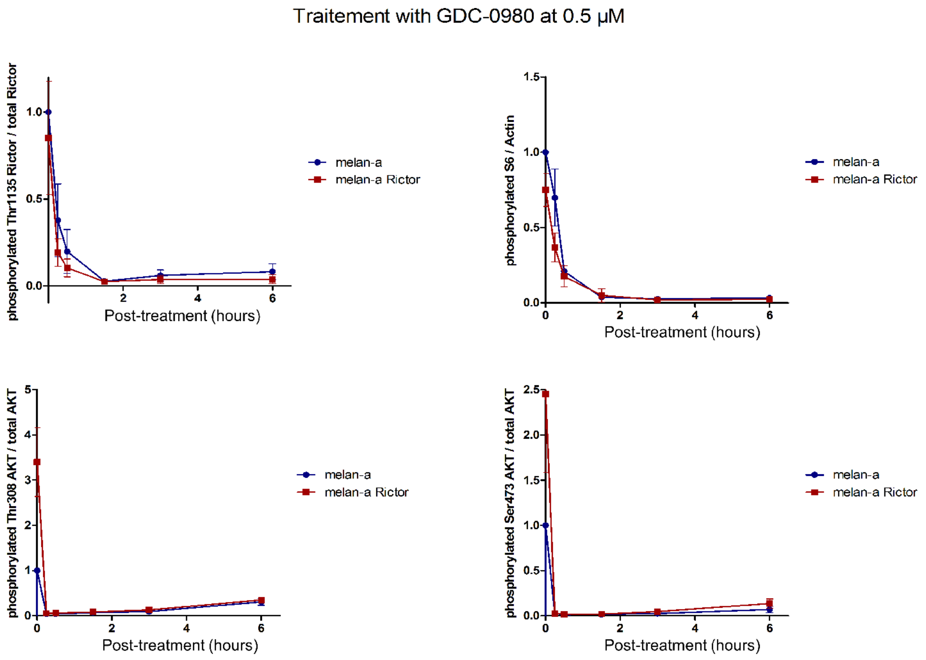

Supplementary Figure S2: Melan-a transfected with RICTOR and controls both have a stable inhibition of AKT Ser473, RICTOR Thr1135, S6 Ser235/236 phosphorylation upon GDC-O980 treatment. Melan-a and Melan-a transfected with RICTOR were treated with DMSO (control 0 min) or GDC-0980 at 5 µM for 15 min, 30 min, 1 h30, 3 h or 6 h. Levels of phosphorylated proteins and total proteins were analyzed by Western Blotting and quantified (data are represented as mean +/− SD).

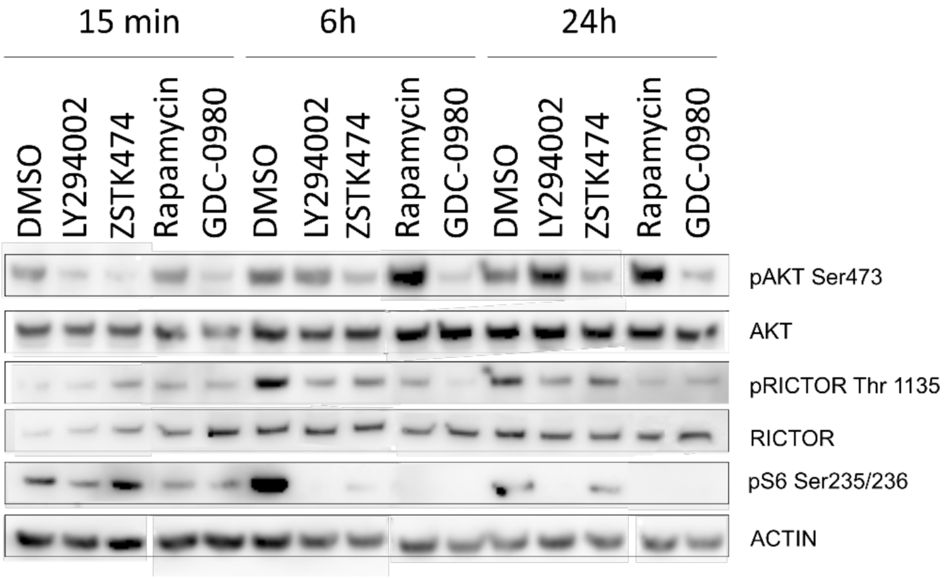

Supplementary Figure S3: PI3K and mTOR inhibitors inhibits of AKT phosphorylation transiently. NHEMs were treated for 15 min, 6 hours or 24 hours with DMSO (control), LY294002, ZSTK474, rapamycin or GDC-0980 at respectively 5 µM, 0.2 µM, 5 µM and 0.5 µM in growing medium. Level of phosphorylated protein or total protein were analyzed by Western Blotting.

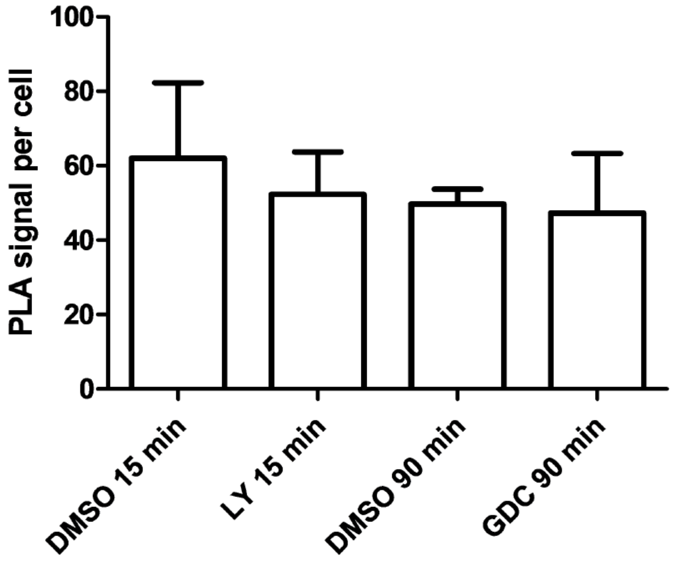

Supplementary Figure S4: Interaction of AKT with RICTOR is independent of their phosphorylation status. Melan-a were treated for 15 min or 1 h30 with LY294002 or GDC-0980. Interaction between RICTOR and AKT was determined by PLA and red dots per cell were quantified (data are represented as mean +/− SD).

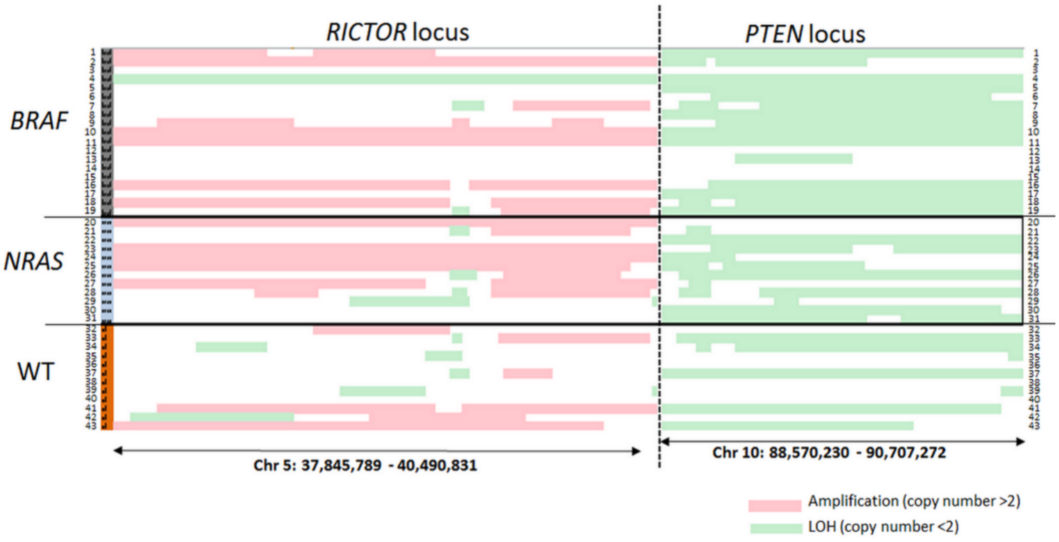

MUTATION	AMPLIFICATION AT RICTOR LOCUS	LOH AT PTEN LOCUS
BRAF	9 /19 (47%)	15 /19 (79%)
NRAS	8 /12 (67%)	7 /12 (58%)
WT	5 / 12 (42%)	5 /12 (42%)
TOTAL	19 /43 (44%)	27 /43 (63%)

Supplementary Figure S5: In BRAF mutated melanoma cell lines, amplification at *RICTOR* locus is associated with LOH at *PTEN* locus. Short term melanoma cell lines were analysed for amplification or deletion at *RICTOR* and *PTEN* loci. Numbers 1 to 43 indicate cell line number. Red lines correspond to amplification (copy number > 2) and green lines to loss of heterozygosity (copy number < 2).

Supplementary Table S1: copy number (CN) details on RICTOR and PTEN loci
